# Childhood cancer presentation and initial outcomes in Ethiopia: Findings from a recently opened pediatric oncology unit

**DOI:** 10.1371/journal.pgph.0003379

**Published:** 2024-07-10

**Authors:** Diriba Fufa Hordofa, Muktar Ahmed, Zewdie Birhanu, Sheila Weitzman, Julie Broas, Aziza Shad, Miguel Bonilla, Thomas B. Alexander

**Affiliations:** 1 Department of Pediatrics and Child health, Jimma University, Jimma, Ethiopia; 2 Department of Epidemiology, Jimma University, Jimma, Ethiopia; 3 Department of Health, Behavior and Society, Jimma University, Jimma, Ethiopia; 4 Hospital for Sick Children, Toronto, Ontario, Canada; 5 The Aslan Project, Inc., Washington, DC, United States of America; 6 Department of Pediatrics, The Herman and Walter Samuelson Children’s Hospital at Sinai, Baltimore, Maryland, United States of America; 7 Department of Global Pediatric Medicine, St. Jude Children’s Research Hospital, Memphis, Tennessee, United States of America; 8 Department of Pediatrics, University of North Carolina, Chapel Hill, North Carolina, United States of America; 9 Department of Pathology and Laboratory Medicine, University of North Carolina, Chapel Hill, North Carolina, United States of America; Rwanda Military Hospital, RWANDA

## Abstract

There were no pediatric oncology centers in southwest Ethiopia prior to 2016. This study aims to describe presenting diagnoses and initial outcomes at Jimma University Medical Center (JUMC), the first pediatric oncology unit (POU) in southwest Ethiopia, provide initial insights into regional pediatric cancer epidemiology, illustrate the rapid growth of pediatric cancer services, and highlight ongoing challenges. We used a retrospective descriptive approach to assess the epidemiologic pattern and initial treatment outcomes of pediatric cancer at JUMC POU from August 2016 through December 2022. During the study period, 749 children were diagnosed with cancer at JUMC. The mean age was 7.2 years (20 days—18 years). Acute lymphoblastic leukemia was the most common diagnosis (16.4%), followed by non-Hodgkin lymphoma (12.4%), Wilms tumor (11.1%), soft tissue sarcoma (8.8%), Hodgkin lymphoma (8.4%), and retinoblastoma (8.3%). Brain tumors accounted for only 2.7% of the diagnoses. Of the 703 patients that were not referred elsewhere, 42% of the patients abandoned treatment, 29% died, 17% completed treatment, and 7% remained on treatment at the time of this assessment. The findings emphasize the growth in the diagnosis and treatment of children with cancer in the southwest region of Ethiopia. The data suggests a different epidemiologic profile of childhood cancer cases diagnosed at the JUMC POU compared to high-income countries and neighbouring countries in Africa. Treatment abandonment remains a barrier to care. Ongoing areas of focus include establishment of a hospital-based cancer registry, reduction of treatment abandonment, improvement of diagnostic capacity, and increased access to advanced supportive care.

## Introduction

More than 80% of childhood cancers are diagnosed in low-income and middle-income countries (LMICs), where access to healthcare is often inadequate [1]. To appropriately plan a health system response to address the burden of childhood cancer, accurate estimates of the disease are important [2]. However, approximately 60% of countries do not have quality population-based cancer registries, and those that do often cover only a small fraction of the population [3]. Ethiopia is the second-most populous country in Africa with estimated 120 million people [4]. However, no population-based data cancer registry exists in Ethiopia except for the Addis Ababa city administration cancer registry, which was established in 2013 [5]. The Addis Ababa cancer registry is focused on adult cancer, leaving the burden and patterns of childhood cancer in Ethiopia unclear. Based on the available data, an estimated 80% of reported cases of cancer are diagnosed at advanced stages, likely due to limited number of oncologists and oncology treatment program in Ethiopia, making it difficult to access cancer treatment services [5].

Although there has been improvement in cancer-related healthcare delivery to children in LMICs overall, the survival rate for children with cancer in many such countries still remains below 30%, in contrast to the overall survival rate of 80% or more observed in high-income countries (HICs) [6]. Factors contributing to the disparity in childhood cancer care and survival rates include lack of access to appropriate cancer care facilities, untimely and inaccurate diagnoses, late presentation of disease for treatment, lack of access to skilled health professionals, high rates of treatment abandonment, and inadequate provision of essential chemotherapy and supportive care drugs [7].

Ongoing global efforts are in place to address this disparity through improving access and quality of childhood cancer care with a goal of achieving a global survival rate of at least 60% for children with cancer by 2030, in accordance with the WHO Global Initiative for Childhood Cancer [8]. Furthermore, international collaborations and partnerships between high resource countries and pediatric units in LMICs have proven to be a successful approach to improve the quality of care and survival for children with cancer [9].

The Aslan Project (Aslan), a US-based non-profit organization, has worked since 2012 to support the initial development and subsequent expansion of pediatric cancer healthcare services and capacity in the country. In 2013, Aslan supported the opening of Ethiopia’s first dedicated pediatric oncology unit (POU) at Tikur Anbessa Specialized Hospital (TASH) in Addis Ababa and launched the country’s first pediatric hematology-oncology (PHO) fellowship program as well as trainings in pediatric oncology nursing and oncology pharmacy. The TASH program now treats more than 700 new cases annually.

A second comprehensive pediatric cancer program was opened at JUMC in August 2016. As the hospital had no prior experience diagnosing or treating children with cancer, Aslan and other partners have conducted numerous capacity building training sessions in pediatric oncology nursing, pharmacy, and pathology throughout the assessment period, both in anticipation of the POU’s opening and to facilitate subsequent growth and sustainability.

Given the dearth of research on the pattern and outcome of childhood cancers in Ethiopia, little is known about the health burden. This poses an immense challenge for health authorities and other stakeholders to prioritize childhood cancer in resource allocation. The study aims to examine the pattern and initial treatment outcomes of childhood cancer among children diagnosed with cancer at JUMC in the first six and half years of its operation. Furthermore, it adds another layer of data to scientific literature on the pattern of childhood cancer in Africa.

## Methods

### Study setting, population and period

The study was conducted at JUMC, located in Jimma City, 352 km southwest of Addis Ababa. Currently, it is the only teaching and referral hospital in southwestern Ethiopia, serving a catchment population of approximately 10 million within three states of the country: mainly Oromia, some parts of South Nation Nationality Population, and Gambella (or sometimes from South Sudan via refugee camps in Gambella). The catchment area has low social economic status and there are several different languages spoken, contributing to the challenge of healthcare delivery. Prior to 2016, there were no options for cancer care at JUMC, no pediatric hematologist/oncologists, no oncology nurses, no oncology pharmacists, no pathology experience with pediatric cancer, no CT or MRI availability, no chemotherapy, no dedicated space, no psychosocial support, and no family support.

### Study design

The investigators applied a descriptive study design, conducting a retrospective review of the medical charts of consecutive children admitted to the JUMC POU from August 11, 2016 (date of first dose of chemotherapy) to December 31, 2022. The study was approval by JUMC ethical review board on 01/19/2018 (#IHRPGD/436/2018). As a retrospective chart review with no reporting of identifiable information, the ethical review board deemed this research was exempt from participant or parent written consent requirement. Clinical records were accessed directly by researchers through 2022 and 2023 to organize this research and analysis. The data collected included patient related characteristics such as age, sex, date of admission, diagnosis, and treatment outcome. During the initial study period, limited data collection, challenges with medical record management, and incomplete follow-up made conducting a meaningful long-term survival analysis difficult.

### Data analysis

Data were analysed using summary measures of descriptive statistics to examine the patterns and outcomes of childhood cancers over the study period. The analyses involved calculating summary statistics, which are now presented to provide an overview of the findings. The pattern of cancer diagnosed was collected from clinical data. Patient status ([1] on treatment [2], dead [3], completed treatment and alive at last follow up, and [4] abandoned treatment and alive at last follow up) was collected and crosschecked from individual patient charts.

### Cancer diagnosis

Bone marrow biopsies were performed by the oncology team, while solid tumor biopsies were performed by surgical subspecialists, depending on the location of the mass. All children with leukemia were diagnosed using morphologic analysis of Wright-Giemsa-stained marrow. Advanced diagnostic techniques such as Cytochemical/immunohistochemical testing, flow cytometry, and cytospin for cerebrospinal fluid analysis were not available. For solid tumors, cancer diagnosis was based on haematoxylin and eosin staining of specimens from a biopsy or fine needle aspirate; if there was undue delay in obtaining histology, a presumptive diagnosis of the likeliest malignancy was made on clinical and imaging grounds. A malignant germ cell tumor diagnosis was supported by measurement of tumor markers (alpha-fetoprotein and beta-human chorionic gonadotropin). Imaging techniques initially included x-ray and ultrasound, with computed tomography scan becoming increasingly available in later years. However, MRI was rarely available during the study period.

### Treatment

During the study period, twelve core treatment regimens were implemented, which were based on protocols developed for Aslan’s initial TASH program. Families were not charged for treatment as it was paid for by a combination of non-profit support and institutional support as there is not a national commitment to cover costs of care for children with cancer yet. However, the supply of chemotherapy remained inconsistent throughout the study period. Intravenous chemotherapy was administered by peripheral IV, as not central access support was available. General surgeons, orthopedic surgeons, ophthalmologists, radiologists, and pathologists were part of the multidisciplinary team. Interventional radiology was not available. Radiation therapy was not routinely available during the first five years, becoming more consistently available for older children during the last year of the study. Availability of antibiotics was limited, with only oral fluconazole and oral acyclovir being the available antifungal and antiviral drugs, respectively. Granulocyte colony-stimulating factors were not consistently available. Transfusion support was significantly limited, primarily restricted to whole blood, while platelets and other blood components were less commonly available.

## Results

### Program development and progress of pediatric hematology-oncology

The JUMC POU was started as part of the development of a comprehensive pediatric cancer program led by Aslan. As previously described, the approach focused on training subspecialty physicians in the Ethiopian setting, rather than external fellowship programs, although each trainee also spent a six month period in a POU outside of Ethiopia with the majority going to a teaching hospital in India [10]. In addition, prior to COVID-19, regular visits to Ethiopia POUs were made by pediatric hematologist/oncologists under the auspices of Aslan. While challenging, this approach established ownership, retained physician leaders in the country, and taught subspecialty care directly within contextual limitations. The first fellow began training at JUMC (then called Jimma University Specialized Hospital) in 2016 under the direction of a full-time international faculty member (MB). The entire spectrum of PHO care needed to be established, including physical ward space, pathology support, radiology support, oncology nursing, oncology pharmacy, chemotherapy access, treatment protocols, multidisciplinary support, infection control, and other supportive care. Through administrative advocacy, external support, and human resource commitment, many of the barriers have been improved.

The first chemotherapy administration for a child in the JUMC POU occurred on August 11, 2016, when a pre-phase with cyclophosphamide and prednisolone were given to an 8-year-old with non-Hodgkin lymphoma. In its early years, the program encountered significant challenges, even to the point of raising questions about the ultimate success and sustainability of the program. During its first two years, the POU was run by one pediatric hematologist-oncologist from El Salvador, and the first cohort of PHO fellows–one from Kenya, and the second from Jimma. The second PHO fellow graduated in 2018 and has remained as faculty at JUMC POU; his consistent leadership and advocacy, as well as Aslan’s sustained support have strengthened the program considerably.

Currently, the JUMC POU has 20 beds in a dedicated space for children with cancer, a pharmacy store, a chemotherapy preparation room, a procedure room, and a playroom. The unit is run by one full-time pediatric hematologist-oncologist, three PHO fellows, ten pediatric oncology nurses, five pharmacists, one psychologist, one social worker, one data clerk, and one nutritionist. A support program run by a local chapter of Tesfa Addis Parents Childhood Cancer Organization (TAPCCO) provides free local housing for up to 20 families as well as nutrition, psychosocial support, transportation, and other services for pediatric patients receiving cancer care and their families.

### Demographic and diagnostic characteristics of pediatric cancer cases at JUMC POU

Of the 749 patients who were treated during the study period, 430 (57.4%) were male. The mean age of patients admitted to the POU was 7 years. The age group of 10–18 years was the most predominant, with 270 patients (36.0%), followed by the age groups of 1–4.99 years (31.5%) and 5–9.99 years (26.0%).

Among the diagnosed childhood malignancies, acute lymphoblastic leukaemia (ALL) was the commonest cancer, accounting for 124 (16.4%) of malignancies, followed by non-Hodgkin lymphoma (NHL) and Wilms tumor, which accounted for 93 (12.4%) and 83 (11.1%) of the cases, respectively (Table 1). The mean ages of ALL, NHL, Wilms tumor, and soft tissue sarcoma patients were 7.6 years, 7.3 years, 3.7 years, and 8.1 years, respectively. Brain tumors as a group only comprised 2.7% of the patients diagnosed at JUMC. Due to limitations in consistent radiographic imaging at diagnosis for most of the study period, staging is not available for the solid tumors.

**Table 1:** Distribution of childhood cancer cases diagnosed at Jimma University Medical Center from August 11, 2016, to December 31, 2022.

	Total		Age (years)					
	Number	Percentage	Mean	0–0.99	1–4.99	5–9.99	10–18	>18
**Acute lymphoblastic leukemia**	123	16.4%	7.6	3	37	38	45	0
**Non-Hodgkin lymphoma**	93	12.4%	7.3	0	32	30	31	0
**Wilms tumor**	83	11.1%	3.7	8	53	16	6	0
**Soft tissue sarcoma**	66	8.8%	8.1	5	13	15	33	0
**Hodgkin lymphoma**	63	8.4%	8.4	0	6	33	24	0
**Retinoblastoma**	62	8.3%	3.0	4	47	10	1	0
**Acute myeloid leukemia**	52	6.9%	8.8	3	6	20	22	1
**Osteosarcoma**	49	6.5%	11.3	0	1	9	38	1
**Neuroblastoma**	38	5.1%	4.7	6	20	3	9	0
**Germ cell tumor**	25	3.3%	8.7	4	3	3	15	0
**Brain Tumor**	20	2.7%	8.1	0	4	9	7	0
**Ewing sarcoma**	17	2.3%	10.7	0	3	2	12	0
**Hepatoblastoma**	16	2.1%	4.4	6	5	1	4	0
**Chromic myeloid leukemia**	12	1.6%	9.1	0	3	3	6	0
**Nasopharyngeal carcinoma**	7	0.9%	13.0	0	0	0	7	0
**Others** *	23	3.1%	8.5	3	5	3	11	1
**Total**	749	100.0%	7.2	42	238	195	271	3

*Others: Three bone tumors not otherwise specified (NOS), three Langerhan’s cell histiocytosis, three pleuropulmonary blastoma, two abdominal masses (NOS), two chondrosarcoma, one mediastinal mass (NOS), one colonic adenocarcinoma, one ganglioneuroma, one hepatocellular carcinoma, one nasopharyngioma, one pancreatoblastoma, one primitive neuroectodermal tumor, one salivary gland carcinoma, one schwannoma, one thyroid carcinoma.

### Treatment status of patients admitted to JUMC POU

Outcomes were categorized as treatment abandonment, death, successful completion of planned treatment, and on treatment. Of the 749 children diagnosed or presumed to have cancer, 46 (6%) were referred to another institution without starting treatment at JUMC. The referred cases include almost all children diagnosed with brain tumors (sixteen cases) and chronic myeloid leukemia (eleven cases), as well as eight children with soft tissue sarcomas, three with acute myeloid leukemia (AML), two with ALL, two with nasopharyngeal carcinoma, one with osteosarcoma, one with Ewing sarcoma, and two others. The JUMC POU is not treating patients with chronic myeloid leukemia because of the lack of diagnostic facilities and drugs needed for such treatment. Other referrals were made due to diagnostic difficulties or a lack of chemotherapy, appropriate surgical services, and the absence of other supportive care products.

**Fig 1:**
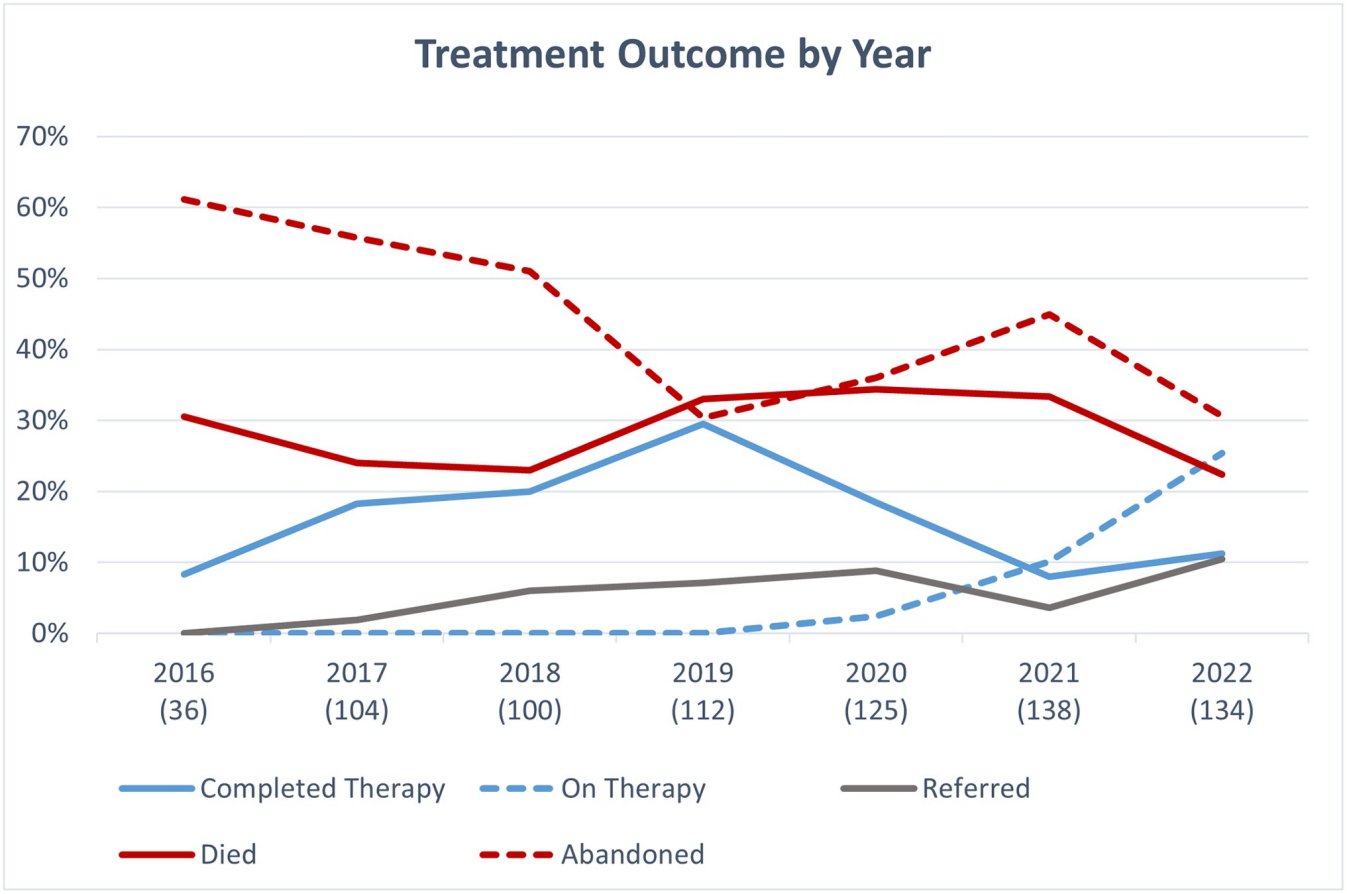
Outcomes by year. Outcome of children with childhood cancer presenting to JUMC, Jimma, Ethiopia by year. Number of new diagnoses per year are listed in parenthesis under the year.

For these reasons, 703 patients during the study period began their cancer management in the POU and are include in the following description of treatment status. A total of 313 (42.0%) children abandoned their treatment and 215 (29.0%) died due to treatment-related toxicity or disease progression. A total of 124 (17.0%) patients successfully completed all their recommended treatment without a known relapse. (Fig 1) Treatment abandonment decreased from 61% in 2016 to 30% in 2019, but then increased in 2020 (36%) and 2021 (45%), likely secondary to effects of the COVID-19 pandemic. Similarly, the rate of treatment completion increased from 8% in 2016 to 29% in 2019, but then decreased during the years of the COVID-19 pandemic.

Out of the 123 patients with ALL, only 9 (7%) completed their planned treatment, while 47 (38%) abandoned treatment and 52 (42%) are known to have died. (Table 2) Thirteen patients (11%) remain on treatment. Of the 93 children diagnosed with NHL, 40 (43%) abandoned treatment, 35 (38%) died, and only 14 (15%) completed their treatment. In contrast, 46% of patients with Hodgkin lymphoma completed treatment without known relapse (Table 2), which is the highest treatment completion rate of all tumor types. Wilms tumor cases had the second highest treatment completion rate at 27%, consistent with the potential for cure with lower intensity therapy in a subset of cases with Wilm’s tumor and Hodgkin lymphoma. The highest documented death rate occurred in patients with AML (48%), consistent with the intensity of therapy as well as the disease related marrow and immune suppression.

**Table 2:** Initial treatment outcomes of children diagnosed with cancer at JUMC in the first 6.5 years of operation.

	Abandoned		Died		Completed Treatment		Referred		On Treatment	
**Acute lymphoblastic leukemia**	47	38.2%	52	42.3%	9	7.3%	2	1.6%	13	10.6%
**Non-Hodgkin lymphoma**	40	43.0%	35	37.6%	14	15.1%	0	0.0%	4	4.3%
**Wilms tumor**	44	53.0%	13	15.7%	22	26.5%	0	0.0%	4	4.8%
**Soft tissue sarcoma**	31	47.0%	14	21.2%	5	7.6%	8	12.1%	8	12.1%
**Hodgkin lymphoma**	19	30.2%	12	19.0%	29	46.0%	0	0.0%	3	4.8%
**Retinoblastoma**	40	64.5%	10	16.1%	7	11.3%	0	0.0%	5	8.1%
**Acute myeloid leukemia**	14	26.9%	25	48.1%	10	19.2%	3	5.8%	0	0.0%
**Osteosarcoma**	23	46.9%	16	32.7%	7	14.3%	1	2.0%	2	4.1%
**Neuroblastoma**	11	28.9%	16	42.1%	7	18.4%	0	0.0%	4	10.5%
**Germ cell tumor**	10	40.0%	9	36.0%	5	20.0%	0	0.0%	1	4.0%
**Brain Tumor**	4	20.0%	0	0.0%	0	0.0%	16	80.0%	0	0.0%
**Ewing sarcoma**	8	47.1%	2	11.8%	2	11.8%	1	5.9%	4	23.5%
**Hepatoblastoma**	7	43.8%	5	31.3%	2	12.5%	0	0.0%	2	12.5%
**Chromic myeloid leukemia**	0	0.0%	1	8.3%	0	0.0%	11	91.7%	0	0.0%
**Nasopharyngeal carcinoma**	3	42.9%	1	14.3%	0	0.0%	2	28.6%	1	14.3%
**Others**	12	52.2%	4	21.7%	5	17.4%	2	8.7%	0	0.0%
**Total**	313	41.8%	215	28.7%	124	16.6%	46	6.1%	51	6.8%

## Discussion

A historic step in the development of pediatric cancer care in Ethiopia occurred on August 11, 2016, when the first dose of a chemotherapeutic agent was administered to a child with cancer in Jimma. Since the initial chemotherapy administration, many challenges have been overcome although many areas of needed growth remain. In this study, we have begun to describe the pattern of pediatric cancers in southwest Ethiopia, by describing cases diagnosed at a new POU over its first six and half years of operation. Cancer care delivery is multidisciplinary, required input and expertise across departments. In fact, certain localized solid tumors can be cured with resection alone, emphasize the important of early recognition and pediatric surgical expertise.

The findings of the present study provide insights into the types of childhood cancer diagnosed in southwest Ethiopia. The most diagnosed type was ALL, followed by NHL and soft tissue sarcoma. These findings differ from other studies conducted sub-Saharan Africa, where NHL has been reported as the most common type, followed by ALL and retinoblastoma [11]. Additionally, in some areas such as Mozambique, Zambia and Malawi, Kaposi sarcoma has been the most commonly reported childhood cancer, although its incidence is linked to HIV prevalence and is shifting [12]. In reporting these findings, it is important to consider the limitations associated with comparisons of hospital-based registries. For example, the presenting diagnosis pattern may be influenced by different patterns in the referral system, traditional healers, or cultural approaches to illness. In particular, only 2.7% of all cases where CNS tumors, which almost certainly reflects misdiagnosis, non-diagnosis, or referral patterns, rather than true epidemiologic differences. However, some of these discrepancies also suggest that epidemiologic profiles of childhood cancer could have significant regional variations. Understanding the local epidemiology of childhood cancer is crucial so that healthcare systems in LMICs can appropriately adapt their resources, infrastructure, and training programs.

Given the side effects and duration of therapy for childhood cancer, active family involvement is required for successful treatment outcomes [13]. Treatment abandonment is the most important cause of childhood cancer treatment failure in sub-Saharan Africa [14–16]. Globally, 99% of treatment abandonment occurs in LMICs [17]. A number of complex reasons for these findings have been identified by previous studies, including financial difficulties, poor access to treatment/health facilities and transportation, and widely-held beliefs of the incurability of childhood cancer [18–20]. Not surprisingly, treatment abandonment likewise was the most common outcome observed for children with cancer treated at the JUMC POU. The high treatment abandonment rate reflected both family and health system challenges during the study period. While not a focus of this study, we informally observed family challenges at JUMC POU such as educational status of parents, family income, distance from home to the treating center, and need to care for the other children in the family. Health system challenges at JUMC POU include limited diagnostics, inconsistent chemotherapy availability, cultural and language barriers, and inconsistent supportive care. The COVID-19 pandemic increased these challenges due to enforced lockdowns, voluntary restrictions, fear of visiting healthcare facilities, and exacerbation of economic challenges for families.

The study also highlights deficiencies in cancer identification and documentation throughout treatment on the POU. First, the limitations of diagnostic techniques and expertise make the diagnosis and reporting of even the most common childhood cancers challenging. While the experience and expertise of JUMC pathologists has increased during the study period, providers continue to rely on morphology alone for all diagnoses. Moreover, for the first several years of the program, there was no dedicated person registering and collecting data on pediatric cancer cases. Since 2018, there has been a dedicated data clerk and there are ongoing efforts to implement a national hospital-based pediatric cancer registry.

The study has several limitations. First, it includes patients visiting the JUMC POU and is not intended to be a population level epidemiologic description. Second, due to the limited diagnostic capacity of the pathology service, data on the specific diagnoses was likely inaccurate in a portion of cases. For example, the number of cases we report of hepatoblastoma, neuroblastoma, and Wilm’s tumor in older children is much higher than expected. Additionally, data on tumor histologic and genomic subtypes is not available. Third, given the limitations on medical record keeping and the lack of a formal hospital-based registry, we are unable to report long term survival data.

Despite these limitations, this report is the first of its kind to describe the pattern and outcome of childhood cancer in this region of Ethiopia. The observed variations in the types of childhood cancer emphasize the need for country-specific approaches and highlight the importance of international collaborations to address the diverse challenges faced by children with cancer worldwide. This research, demonstrating the critical needs in diagnostics, hospital-based registries, chemotherapy access, and supportive care.

Overall, these findings add to the growing experience of treating childhood cancer in Ethiopia and will aid the efforts of program planners and policy makers such as the Federal Ministry of Health. The importance of the programmatic growth in purely human terms is, of course, incalculable, but that is not the only advantage to establishing pediatric cancer care and improving survival from pediatric cancers. Two recent publications discussed the cost-effectiveness of treating pediatric cancer, one in Ethiopia and the second in Uganda [21, 22]. Both concluded that, when the cost of the loss of years of productive life was weighed against the total cost of running a pediatric cancer unit, establishing and paying for a quality POU in LMICs would prove to be a highly cost effective intervention for governments.

This study follows pediatric cancer treatment from August 11, 2016, to December 31, 2022 at JUMC, a public health facility in southwestern Ethiopia, more than 350 km from the capital Addis Ababa. The JUMC POU opened in 2016 with no experience in, or services for, children with cancer. The unit has since provided more than 700 patients with multidisciplinary care, with growing psychosocial family support. Despite the high-level of treatment abandonment, temporally exacerbated by the COVID-19 pandemic, a noteworthy percentage of children in this newly opened POU have completed their treatment regimen or are on active treatment, and certain remissions have been achieved while the clinical care delivery continues a dynamic phase of growth and improvement.

## Supporting information

S1 Table Data supporting Fig 1. Outcome of children with childhood cancer presenting to JUMC, Jimma, Ethiopia by year. Number of new diagnoses per year are listed in parenthesis next to the year.
(XLSX)
